# Pd-Shaped Nanoparticles Modified by Gold ad-Atoms: Effects on Surface Structure and Activity Toward Glucose Electrooxidation

**DOI:** 10.3389/fchem.2019.00453

**Published:** 2019-06-26

**Authors:** Thibault Rafaïdeen, Stève Baranton, Christophe Coutanceau

**Affiliations:** Catalysis and UnConventional Media group, IC2MP, Université de Poitiers, UMR CNRS 7285, Poitiers, France

**Keywords:** ad-atoms, glucose electrooxidation, palladium, gold, nanocubes, nanooctahedrons, nanospheres

## Abstract

Palladium nanoparticles (Pd-NPs) with controlled distributions of sizes and shapes (nanospheres–Pd-NS-, nanocubes -Pd-NC-, and nanooctahedrons -Pd-NO-) are synthesized by wet chemistry methods and characterized by TEM/HRTEM. The surfaces of Pd-NPs are modified by spontaneous adsorption of gold and characterized by cyclic voltammetry in acidic medium. It is shown that the modification of Pd-NPs by dipping in HAuCl_4_ solutions of different concentrations allows controlling the surface coverage by gold. It is also shown that the modification of Pd-NPs surfaces involves first the formation of PdAu surface alloys. For higher coverages, both PdAu surface alloys and pure Au structures are formed. The activity toward the glucose electrooxidation reaction is determined by linear scan voltammetry (LSV). Higher activity is observed on pure Pd-NC presenting extended (100) surfaces than on Pd-NO with mainly (111) surface orientation and on Pd-NS without preferential surface orientation, both these latter Pd-NPs displaying almost the same activity. The modification of the surface by spontaneous adsorption of gold greatly improves the activity of all Pd-NPs. However, Au-modified Pd-NC materials remain the most active catalysts. PdAu surface alloys seem to be involved in the improvement of the catalytic activity at low potentials, although the role of pure gold structures on Pd-NPs toward the enhancement of the catalytic activity cannot be excluded for high gold coverage. The study allows a better understanding of the material structure/electrocatalytic behavior relationship.

## Introduction

The selective conversion of aldoses such as glucose and xylose into valuable carboxylates/carboxylic acids represents an extremely attractive way to produce bio-sourced building blocks for fine chemistry. Indeed, xylonic and gluconic acids belong both to the top-30 list of value-added chemicals from biomass (Werpy et al., 2004), which can be used in numerous applications, such as building blocks for the syntheses of various biodegradable chemicals, chelating agents, additives for cement, concrete, food, and pharmaceutical (Liu et al., 2012; Cañete-Rodríguez et al., 2016).

In an industrial point of view, heterogeneous (electro)catalysis processes can be excellent alternatives to classical biotechnological processes for the conversion of aldoses into carboxylates because these methods allow achieving higher activity (reaction rate) (Tathod et al., 2014). However, the solid catalysts and the heterogeneous catalytic processes must also allow achieving selectivity as high as those provided by classical biotechnological processes and higher stability (Climent et al., 2011). Gold (Baatz and Prüße, 2007; Bujak et al., 2012), palladium (Liang et al., 2008) and alloyed Au-Pd materials (Hermans et al., 2011) are known to be good catalysts for the active and selective oxidation of glucose into gluconate. These materials have also displayed good electroactivity toward glucose electrooxidation, as monometallic (Becerik and Kadirgan, 1992; Pasta et al., 2010) or bimetallic nanostructures (Brouzgou and Tsiakaras, 2015). Yan et al. (2014) have studied the electrocatalytic behavior of non-alloyed Pd-Au/C catalysts and found higher poisoning tolerance during glucose oxidation with a Pd_3_-Au_7_ atomic composition. More recently, Rafaïdeen et al. (2019) obtained high conversion rate of glucose and high selectivity toward gluconate (higher than 85%) at low anode potential (ca. 0.4 V vs. RHE) with an alloyed Pd_3_Au_7_ (bulk atomic composition) nanocatalyst dispersed on a carbon powder as support. These authors determined the global surface composition as well as the composition of the PdAu surface alloys and the amount of isolated Au surface structures (unalloyed gold). They observed that the composition of the nanoparticle surface was different to the one of the nanoparticle bulk, and that the surface composition was the key parameter ruling the activity and selectivity of the catalyst. Therefore, a better understanding of the structure/surface composition/electrocatalytic behavior relationship is essential on a fundamental point of view for the determination of reaction mechanisms and on a practical point of view for the rational design of active and selective catalysts.

Real Pd catalytic nanoparticles are mainly composed of (100) and (111) surface nanodomains, consequently, the current study on Pd nanospheres (Pd-NSs), nanocubes (Pd-NCs), and nanooctahedrons (Pd-NOs) serves as a link between the electrocatalytic behavior of low-Miller-index Pd single crystals and real Pd nanoparticles (Pd-NPs). Moreover, it has been established in the literature that the modification of Pd by gold led to improve the activity toward glucose electrooxidation. Then, it appears of paramount importance to investigate Au-modified shaped Pd-NPs to gain insights on the structure/surface composition/electrocatalytic behavior relationship. A method consists then in synthesizing Pd nanoparticles with controlled shape and size distributions, modifying their surfaces by ad-atoms and studying their electrochemical behavior. Indeed, the electrochemical properties of nanocrystals are not only determined by the large proportion of surface atoms but also by their crystallographic arrangement at the particle surface (Henry, 2003, 2005). Moreover, Zalineeva et al. (2013) and Wang et al. (2017) showed that the modification of Pd-NC by bismuth ad-atoms led to increase the catalytic activity toward glycerol electrooxidation.

For metals crystallizing in a face centered cubic (fcc) structure, such as platinum, palladium, and gold, the relationship between the shape of the nanoparticles (NPs) and the main surface orientation are well-established (Kim et al., 2004, 2014; Song et al., 2005; Solla-Gullón et al., 2008). Pd-NCs present mainly (100) surface domains, Pd-NOs exhibit mainly (111) surface domains and Pd-NSs have no preferential surface orientation. Because Pd-NSs, Pd-NCs and Pd-NOs serves as a link between the electrocatalytic behavior of low-Miller-index Pd single crystals and real Pd-NPs, the morphologies of the shaped catalysts will be characterized by TEM/HRTEM and their electrocatalytic activity will be compared. Further, the effects of modification of the surface of Pd-NPs with narrow size and shape distributions by spontaneous adsorption of gold ad-atoms will be studied. The surface microstructures will be investigated by cyclic voltammetry (CV) in an acidic electrolyte. The electrocatalytic behavior of the shaped Pd-NPs, unmodified and modified by gold ad-atoms, toward glucose electrooxidation will tbe investigated in an alkaline medium.

## Experimental

### Syntheses and Characterization of Shaped Pd Nanoparticles (Pd-NPs)

The colloidal methods developed by Xia et al. (Lim et al., 2009; Shao et al., 2013) is slightly modified to synthesize shaped Pd-NPs (Zalineeva et al., 2015b).

Pd-NCs presenting mainly (100) surface orientation are prepared as follows. A first solution (sol. 1) of 0.06 g ascorbic acid (reagent grade, Sigma-Aldrich) as reducing agent in 4 mL ultrapure water (MilliQ®, Millipore, 18.2 MΩ cm) and a second solution (sol.2) containing 0.3 g potassium bromide (KBr, 99.95%, Aldrich) as capping agent, 0.1 g polyvynilpyrrolidone (PVP, Mw ≈ 55,000, Sigma-Aldrich) and 0.062 g potassium tetrachloropalladate (K_2_PdCl_4_, 99.99%, Alfa Aesar) in 7 mL ultrapure water are first prepared and allowed resting for 30 min. A glass reactor is heated up to 80°C in a heating bath. Sol. 2 is first put in the glass reactor at 80°C and then sol. 1. The reaction is performed at 80°C for 3 h until the mixture becomes dark brown or black. Then the heating is stopped, and the temperature is allowed decreasing down to room temperature (20°C).

Pd-NOs presenting mainly (111) surface orientation are prepared by slightly modifying the above-described protocol. In the present case, the palladium salt (0.063 g) is first dissolved in 3 mL ultrapure water/ethanol mixture (2/1 in volume). Then PVP (0.1 g) is added (sol.3). The fourth solution (sol.4) contains 0.18 g citric acid (reagent grade, Sigma-Aldrich) in 8 mL ultrapure water/ethanol mixture (3/1 in volume). Citric acid acts as reducing agent and citrate ions act as capping agent instead of bromide ions. Both latter solutions are introduced in the reactor beforehand heated at 80°C and the same protocol as for Pd-NC is applied.

At last, Pd-NS without preferential orientation are synthesized using the same protocol as for Pd-NC, but in the absence of KBr.

The removal of PVP is performed by diluting the colloidal solution in 250 mL of ultrapure water. The surfactant is dissolved in the aqueous solution and the addition of NaOH pellets leads the NPs to settle down at the bottom of the beaker (Vidal-Iglesias et al., 2012). After sedimentation of Pd-NPs, the supernatant liquid is gently removed using a micropipette. The protocol is repeated several times until the cleanliness of the NPs, as determined by cyclic voltammetry in sulfuric acid electrolyte (see below), was judged acceptable (when redox peaks assigned to surface reactions on (100) and (111) are well-defined and do not change with further cleaning steps).

The shapes of the Pd-NPs are monitored by TEM/HRTEM using a JEOL JEM 2100 (UHR) microscope with a resolution of 0.19 nm. The mean sizes of the Pd-NPs are evaluated considering the length of the longer edge for Pd-NC, the tip-to-tip length for Pd-NO (Zalineeva et al., 2017) and the diameter for Pd-NS, by counting between 200 and 300 isolated NPs using the ImageJ free software (Rasband, 2009) for each Pd-NPs sample, in order to obtain acceptable statistical samples.

The surface of Pd-NPs is modified by spontaneous adsorption of Au ad-atoms by dipping for 1 min without potential control the Pd electrodes into aqueous (ultrapure water) solutions of different concentrations in auric acid (HAuCl_4_·3 H_2_O, 99.99 %, Alfa Aesar) from 0.01 to 1.00 mol L^−1^.

### Electrochemical Measurements

The working electrode consists in a 3.14 mm^2^ disc gold substrate (99.95 % purity, Alfa Aesar) on which a 3 μL aqueous droplet containing ca. Fourteen microgram of unsupported Pd nanoparticles are dipped and dried under nitrogen flow (U-quality, Air Liquide) before cyclic and linear scan voltammetry (CV and LSV, respectively) measurements.

The electrolytes for electrochemical experiments are prepared from ultrapure water: 0.5 M H_2_SO_4_ (suprapur, Merk) N_2_-purged aqueous solution for surface structure evaluation by CV or 0.1 M NaOH (Semiconductor Grade 99.99%, Aldrich) N_2_-purged aqueous solution for 0.1 M glucose oxidation reaction by LSV. The reference electrode is a reversible hydrogen electrode (RHE) and the counter electrode is a 3 cm^2^ glassy carbon plate. The cleanliness of the Pd-NPs surface is checked by recording CVs from 0.100 to 0.600 V vs. RHE in acidic medium. The surface structure of Pd-NPs modified by gold ad-atoms was checked by recording CVs between 0.100 and 1.450 V vs. RHE at a scan rate of 5 mV s^−1^ and at 293 K.

## Results and Discussion

### Transmission Electron Microscopy Results

**Figure 1:**
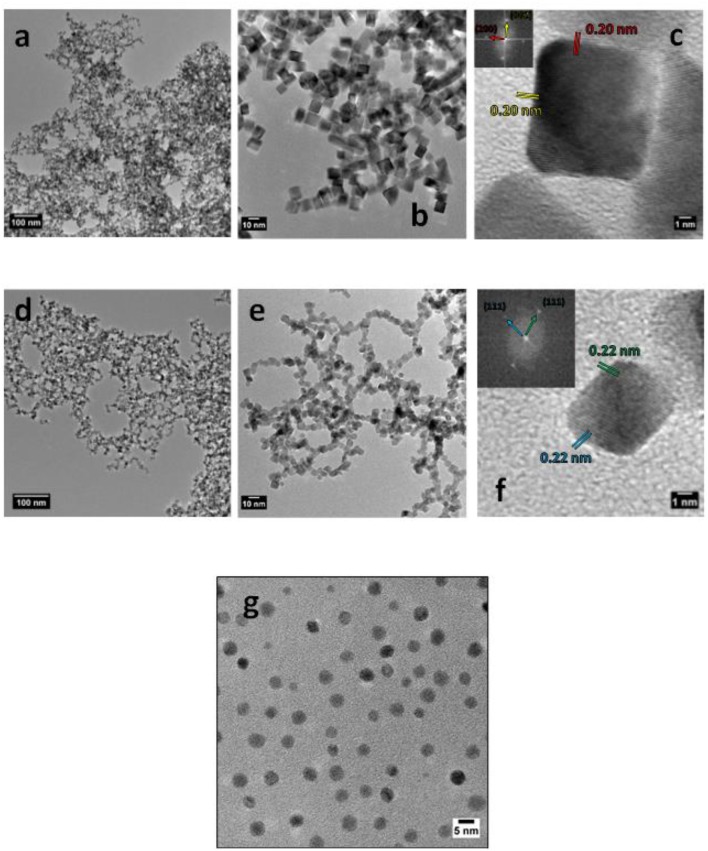
TEM images with different magnifications for **(a–c)** Pd nanocubes, **(d–f)** Pd nanooctahedrons, **(g)** Pd nanospheres. Inset in **(c, f)**: FFT patterns with corresponding crystallographic orientation, from which d-spacing were determined.

The colloidal synthesis methods used in the present work are expected to provide samples of Pd-NCs, Pd-NOs and Pd-NSs. TEM images in Figure 1 confirm the expected results. Typical micrographs with different magnifications in Figures 1a–c present very high density of square projections which correspond to the formation of cubic shaped nanoparticles (Figures 1a,b). This means that Pd-NCs will exhibit mainly (100) surface domain orientation (Shao et al., 2013). This is confirmed by the FFT pattern calculated from HRTEM micrograph (Figure 1c), from which a d-spacing of 0.20 nm corresponding to (200) interplanar distance has been determined in the direction of cube faces. The mean edge length of the Pd-NCs is found to be close to 10 nm.

Micrographs in Figures 1d–f show very high density of projections with mainly rhombohedral shapes, with well-defined angles and edges indicating the formation of facetted nanoparticles. TEM images also reveal that the Pd-NOs are often truncated at their extreme ends. The main Pd-NPs shape is therefore truncated octahedron (Figures 1d,e), which will exhibit mainly (111) preferential surface domain orientation (Shao et al., 2013). This is confirmed by the FFT pattern calculated from HRTEM micrograph (Figure 1f), from which a d-spacing of 0.23 nm corresponding to (111) interplanar distance has been determined in the direction of octahedron faces. The mean tip-to-tip length of the projections is found to be ca. 7 nm.

At last, micrographs in Figure 1e show projections with mainly circular shapes, indicating the formation of spherical particles. A mean diameter of ca. 4 nm is estimated. In this case, no well-defined faces or angles are observed indicating that the surface of these particles displays no preferential orientation and is composed of very small coherent surface domains separated by surface defects.

### Electrochemical Characterization

**Figure 2:**
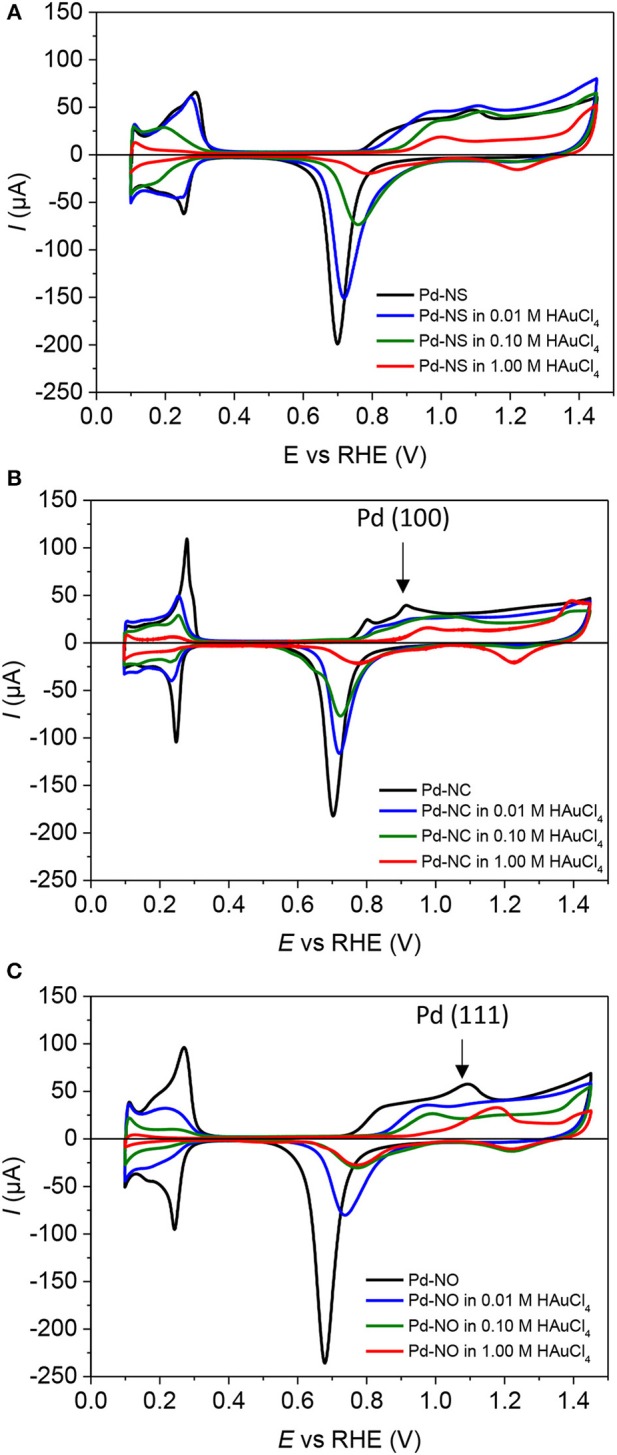
CV profiles in the 0.10–1.45 V range obtained in N_2_-purged aqueous H_2_SO_4_ 0.5 M electrolyte (T = 293 K, scan rate = 5 mV s^−1^) for **(A)** Pd-NS **(B)** Pd-NC, and **(C)** Pd-NO. – for pure Pd-NPs, – after dipping 1 min in HAuCl_4_ 0.01 M, – after dipping 1 min in HAuCl_4_ 0.10 M and – after dipping 1 min in HAuCl_4_ 1.00 M.

CV profiles recorded in 0.5 M H_2_SO_4_ electrolyte between 0.100 V and 1.450 V vs. RHE on the different Pd-NPs and Au-modified Pd-NPs are given in Figures 2A–C for Pd-NSs, Pd-NCs and Pd-NOs, respectively. For the sake of clarity, only the CVs of Pd-NPs modified by dipping the electrodes 1 min in 0.01 M, 0.10 M, and 1.00 M HAuCl_4_ aqueous solutions are presented. Here, it is important to underline that under the experimental conditions of the electrochemical measurements, the potential domain of underpotential deposited H (H_UPD_) and desorption of H_UPD_ is separated from that of H absorption and desorption of absorbed H (Zalineeva et al., 2015b, 2017).

Regarding the unmodified Pd-NPs, the CV profiles reveal cathodic and anodic peaks that correspond to H_UPD_ and desorption of H_UPD_ in the 0.100 to 0.400 V vs. RHE potential region and to surface oxide formation/reduction in the 0.500 to 1.450 V vs. RHE potential region. In the potential region of surface oxide formation, the sharp anodic peak at ca. 0.900 V vs. RHE for Pd-NCs (Figure 2B) is related to the oxidation of (100) surface domains, whereas that at 1.100 V vs. RHE for Pd-NOs (Figure 2C) is related to the oxidation of (111) surface domains (Hoshi et al., 2006; Hara et al., 2007). In the hydrogen region, Pd-NCs also leads to sharper H_UPD_ reversible redox peaks at ca. 0.280 V (positive current)/0.250 V (negative current) vs. RHE than Pd-NOs and Pd-NSs, and it can be reasonably proposed that these features are related to H adsorption/desorption on (100) Pd surface nanodomains. Intense shoulders at ca. 0.200 V vs. RHE are only observed for Pd-NOs, that can be reasonably assigned to H adsorption/desorption on (111) Pd surface nanodomains. Both intense peaks at higher potentials, located at 0.270 V vs. RHE (oxidation peak) and 0.240 V vs. RHE (reduction peak) are slightly shifted toward lower potentials than in the case of Pd-NCs. Those peaks likely contain the contributions of hydrogen adsorption/desorption on Pd (111) surface domains and those, lower than in the case of Pd-NC but still present, of hydrogen adsorption/desorption on Pd (100) surface domains. This observation confirms the truncation of Pd-NOs observed by TEM. At last, Pd-NSs lead to the same features as for Pd-NCs and Pd-NOs but with broader peaks displaying lower relative intensities in the potential region of H adsorption/desorption, whereas in the potential region of Pd surface oxidation the peaks at ca. 0.900 V and 1.100 V vs. RHE are no more clearly visible (Figure 2A). The CV profiles recorded on Pd-NCs and Pd-NOs strongly resemble those obtained on (100) and (111) single crystals (Hoshi et al., 2002; Hara et al., 2007), whereas that recorded on the Pd-NSs is similar to those recorded on highly stepped (100) and (111) Pd surfaces (Hoshi et al., 2002).

In the case of Pt-shaped NPs, a method for the determination of the (100) and (111) surface domain ratios based on spontaneous adsorptions of bismuth and germanium can be found in the literature (Rodríguez et al., 2005a,b; Solla-Gullón et al., 2008). Such a method was not yet developed for the surface characterization of shaped Pd-NPs surfaces. Therefore, we can only conclude qualitatively on the preferential presence or not of (100) and (111) surface domains on Pd-NPs from TEM/HRTEM and from particular electrochemical features in CVs.

**Figure 3:**
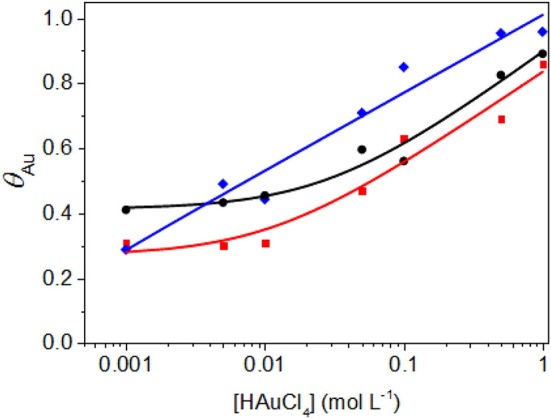
Plot of the Au coverage (θ_Au_) after dipping in HAuCl_4_ solution at T = 293 K as a function of the HAuCl_4_ concentration. – for Pd-NS, – for Pd-NC and – for Pd-NO.

The modification of the surface of Pd-NPs by spontaneous adsorption of gold translates into important changes in the CV profiles. Considering the H adsorption/desorption potential region, the charges involved for H underpotential deposition and desorption of H_UPD_ decreases with the increase of the auric acid concentration in the dipping solution; this trend indicates that the gold surface coverage also increases. The redox peaks at ca. 0.200 and 0.260 V vs. RHE observed on Pd-NOs and Pd-NCs, respectively, disappear for a very low auric acid concentration (0.01 M), indicating that Pd(111) and Pd(100) surface domains are covered by gold (Figures 2B,C). The determination of the charge for H_UPD_ desorption from the Pd surface was used to evaluate the Au coverage (θ_Au_) over the potential range from 0.100 to 0.380 V vs. RHE after correction of the capacitive current measured at ca. 0.400 V vs. RHE (in the double layer potential region). Figure 3 displays the Au surface coverage as a function of the auric acid concentration in the dipping solution. The coverage of the Pd surface increases with the auric acid concentration. The affinity of Au ad-atoms seems higher for Pd(111) surface domains and/or for defects (edges, corners, steps, etc.) than for Pd(100) surface domains, as higher Au coverage are obtained for Pd-NOs and Pd-NSs than for the Pd-NCs.

In the region of Pd surface oxide formation, between 0.700 and 1.450 V vs. RHE, the modification of Pd surface by Au ad-atoms leads first to the disappearance of the oxidation peaks at ca. 0.900 V vs. RHE (Figure 2B) and ca. 1.100 V vs. RHE (Figure 2C) assigned to the oxide formation on Pd(100) and Pd(111) surface domains, respectively, even for low Au coverage. This confirms that surface domains are preferentially covered by gold ad-atoms. The second remark is that the partial coverage of the Pd surface by gold ad-atoms delays the surface oxidation as this latter process starts at higher potentials in all cases (Figures 2A–C). The third remark is that the charge of the surface oxide reduction peak in the 0.600 to 0.900 V vs. RHE range decreases with the increase of the auric acid concentration in the dipping solution, confirming the increase of the Pd surface coverage by gold ad-atoms. More intriguing is the position shift of this reduction peak with the increase of Au surface coverage. According to Rand and Wood (Rand and Woods, 1972), the shift of the reduction peak position is due to the formation of PdAu surface alloys, and the position of the reduction peak potential ($\left(E_{\text{p}}^{\text{Alloy}}\right)$ is directly related to the composition of the PdAu surface alloy. This composition can be calculated using the following equations (Simões et al., 2009; Lankiang et al., 2015):

(1) $$\begin{array}{rcl} E_{\text{p}}^{\text{Alloy}} & = & {X_{\text{pd}}E}_{\text{p}}^{\text{Pd}}+X_{\text{Au}}E_{\text{p}}^{\text{Au}} \end{array}$$

(2) $$\begin{array}{rcl} \Rightarrow \;X_{\text{Pd}} & = & \frac{E_{\text{p}}^{\text{Alloy}}-E_{\text{p}}^{\text{Au}}}{E_{\text{p}}^{\text{Pd}}-E_{\text{p}}^{\text{Au}}} \end{array}$$

(3) $$\begin{array}{rcl} X_{\text{Au}} & = & 1-X_{\text{Pd}} \end{array}$$

where $E_{p}^{\text{Au}}\;$and $E_{\text{p}}^{\text{Pd}}$ are the reduction peak positions for pure Au (1.221 V vs. RHE) and pure Pd (0.699, 0.702, and 0.680 V vs. RHE for Pd-NSs, Pd-NCs, and Pd-NOs, respectively), respectively, and *X*_Au_ and *X*_Pd_ are the Au and Pd atomic ratios in the PdAu surface alloy, respectively.

The appearance and growth with the Au-coverage of the reduction peak centered at 1.221 V vs. RHE indicates the formation of non-alloyed gold structures at the surface of Pd-NPs. This peak is typical of the reduction of gold surface oxide formed for the incursion of electrode in region of potentials higher than 1.300 V vs. RHE, and can be used to determine the real electrochemical surface area of the deposited pure gold structures. From the determination of the surface composition, the real electrochemical surface of Au-modified Pd-NPs can be determined according to the following equations (Łukaszewski and Czerwinski, 2010; Mougenot et al., 2011):

(4) $$\begin{array}{rcl} S_{\text{Au}} & = & \frac{Q_{\text{Red}}^{\text{AuO}}}{Q_{\text{Red},\text{theo}}^{\text{AuO}}} \end{array}$$

(5) $$\begin{array}{rcl} S_{\text{PdAu}} & = & \frac{Q_{\text{Red}}^{\text{PdAu}\left(\text{O}\right)}}{Q_{\text{Red},\text{theo}}^{\text{PdO}}X_{\text{Pd}}+Q_{\text{Red},\text{theo}}^{\text{AuO}}X_{\text{Au}}} \end{array}$$

where $Q_{\text{Red}}^{\text{AuO}}$ and $Q_{\text{Red}}^{\text{PdAu}\left(\text{O}\right)}$ are the charges in the Au and PdAu reduction peaks corrected from the capacitive current, respectively, and $Q_{\text{Red},\text{theo}}^{\text{PdO}}$ and $Q_{\text{Red},\text{theo}}^{\text{AuO}}$ are the theoretical charge values for the desorption of an oxide monolayer from smooth and flat Pd and Au surfaces, respectively. The upper limit potential of 1.450 V vs. RHE in 0.5 M H_2_SO_4_ electrolyte corresponds to that for the formation of a PdO monolayer (Grdén et al., 2008), and under these conditions $Q_{\text{Red},\text{theo}}^{\text{PdO}}$ = 424 μC cm^−2^. For gold, Kahyaoglu (Kahyaoglu, 1981) determined the quantity of electricity associated with the reduction of Au surface oxide as a function of the upper potential limit in 0.5 M H_2_SO_4_ electrolyte, and found a value of 270 μC cm^−2^ for an upper potential limit of 1.450 V vs. RHE.

The first conclusion is that very high Au coverage, between 80 and 90%, can be achieved. The second one is that the kinetics of Au spontaneous deposition and of surface coverage are higher on Pd-NOs than on other Pd-NPs, Pd-NCs showing the lowest ability toward this modification. The third one is that the deposition of gold ad-atoms on Pd-NPs consists not simply in adsorbed Au atoms on the Pd surfaces, but also in the insertion of part of gold atoms into the first Pd atom rows to form PdAu surface alloys. The composition of the Pd surface alloys are calculated from Equations (1–3) after fitting of the reductions peaks using bi-Gaussian curves, while real electrochemical surface areas of PdAu surface alloys and isolated pure gold structures are determined from Equations (4, 5); results are given in Table 1. Under the experimental conditions used in the present work, the gold content in the surface alloys increases with the increase of the auric acid concentration in the dipping solution from 0.01 to 1.00 M. For low auric acid concentration, the formation of PdAu surface alloys is predominant, and no or few (not detectable) non-alloyed gold structures are formed. As the concentration of auric acid is increased, the ratio between the pure gold surface and the PdAu surface alloys increases. This suggests that part of Au atoms are only deposited on the Pd-NPs surface without forming any alloy structure for high auric concentrations.

**Table 1:** Composition of PdAu surface alloys, real electrochemical surface area of PdAu surface alloys (*S*_PdAu_), real electrochemical surface area of Au (*S*_Au_), and total real electrochemical surface area (*S*_Total_ = *S*_PdAu_+ *S*_Au_) for the Au-modified Pd-NPs.

	**C_**HAuCl4**_ (mol L-1)**	**Pd/Au alloy comp (at%)**	***S*_**PdAu**_ (cm^**2**^)**	***S*_**Au**_ (cm^**2**^)**	***S*_**Total**_ (cm^**2**^)**
Pd-NS	0.01	96/4	6.46	0.00	6.46
	0.10	88/12	5.07	0.04	5.11
	1.00	62/38 86/14	0.39 1.19	1.00	2.58
Pd-NC	0.01	98/2	6.95	0.00	6.95
	0.10	96/4	2.96	0.08	3.04
	1.00	50/50 86/14	0.10 1.24	1.34	2.68
Pd-NO	0.01	94/6	5.61	0.00	5.61
	0.10	68/32 86/14	0.64 1.90	0.84	3.38
	1.00	64/36 86/14	0.12 1.65	0.70	2.47

It is also worth to note that for high Au coverage, two different surface alloys (with different compositions) are obtained (appearance of two reduction peaks between 0.700 and 1.200 V vs. RHE). All Pd-NPs samples display a first surface alloy composition of Pd 86 at%/Au 14 at%, i.e., close to Pd_6_Au surface structure, which could correspond to a thermodynamically stable Pd_3_Au bulk structure (Okamoto and Massalski, 1985). Amongst the PdAu surface alloys formed, the one with this composition represents by far the main contribution to the whole electrochemical real surface area.

The formation of PdAu surface alloys can occur directly during the Au ad-atoms deposition process or after cycling up to 1.45 V vs. RHE involving a place exchange reaction promoted by surface oxide formation. This aspect is discussed in the subsection Electrocatalytic Behavior Toward Glucose Oxidation from electrocatalytic behaviors of PdAu nanocatalysts.

### Electrocatalytic Behavior Toward Glucose Oxidation

Polarization curves (corresponding to the third stable CV between 0.050 and 0.750 V vs. RHE) for the electrooxidation of 0.1 M glucose have been recorded in 0.1 M NaOH electrolyte on pure Pd-NPs and on Pd-NPs modified by Au spontaneous deposition.

**Figure 4:**
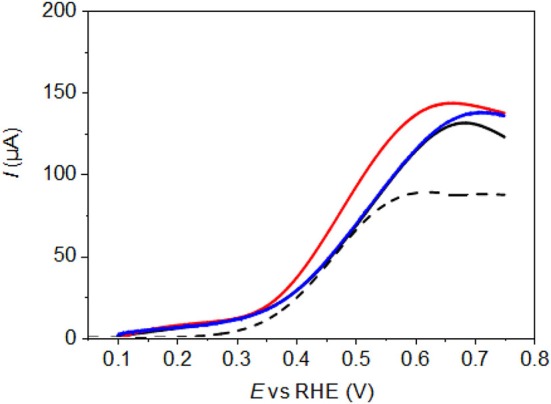
Polarization curves recorded for the electrooxidation of glucose 10^−1^ mol L^−1^ in NaOH 10^−1^ mol L^−1^ aqueous electrolyte (T = 293 K, scan rate = 5 mV s^−1^) on unmodified Pd-NPs. – for Pd-NS, – for Pd-NC and – for Pd-NO; - - activity of pure Au substrate.

Because, all electrodes are loaded with the same mass of Pd-NPs, Figure 4 compares the mass activity, in terms of current density as a function of the electrode potential, of Pd-NSs (black line), Pd-NCs (red line) and Pd-NOs (blue line). The polarization curve for glucose oxidation on the pure gold substrate is also given in Figure 4, and it can be seen that it shows relatively important activity from ca. 0.3 V vs. RHE. However, it is likely that the real contribution of the substrate, if any, to the current recorded on dense Pd-NPs thin films (a black film of Pd-NPs is formed on the surface of the gold substrate) is much lower due to the surface coverage (shadowing of the Au substrate) and glucose consumption at Pd nanoparticles (depletion of the glucose concentration at the Au substrate). Experiments were performed by depositing less Pd nanoparticles on the Au substrate. The Au surface oxide formation and reduction features between 1.20 and 1.45 V vs. RHE become visible, which is not the case under the conditions used in the present case (Figure 2). If the electrolyte does not reach the gold substrate, therefore, it can be concluded that the gold substrate does not participate to the current recorded for the glucose electrooxidation.

Anyway, it is clear that the Pd-NC material presenting defined (100) surface domains leads to higher activity than the Pd-NO one with preferential (111) surface orientation and the Pd-NS one without preferentially oriented surface, as higher current densities are recorded from the onset potential of ca. 0.300 V vs. RHE. In the potential range from 0.300 V to 0.550/0.600 V vs. RHE, Pd surfaces are not expected to adsorb OH species, and shaped Pd-NPs has been shown to be active for polyol oxidation only for potentials higher than 0.600 V vs. RHE (Zalineeva et al., 2013, 2015a). Therefore, the glucose oxidation pathway doesn't involve a Langmuir-Hinshelwood like mechanism, which stipulates the adsorption of organic species on a surface site, of hydroxyl species on another surface site, surface reaction between both adsorbed species and desorption of the final product. Previous studies of glucose oxidation on platinum indicated that adsorbed glucose was transformed into gluconolactone through dehydrogenation reaction at low and intermediate potentials, which after desorption from the surface could be hydrolyzed into gluconate by hydroxyl ions in the alkaline electrolyte (Ernst et al., 1979; Lei et al., 1995; Beden et al., 1996). We reasonably propose that the same mechanism occurs at Pd surfaces, owing to the similarity in catalytic behavior of both Pt and Pd metals (Coutanceau et al., 2019). Therefore, the higher activity of Pd-NCs in the 0.300 to 0.750 V vs. RHE potential range is related to the higher ability of (100) surface domains to adsorb glucose and/or to desorb gluconolactone. It is worth to note that similar observation was made by Zalineeva et al. (2015a) for the electrooxidation of glycerol on shaped Pd-NPs.

**Figure 5:**
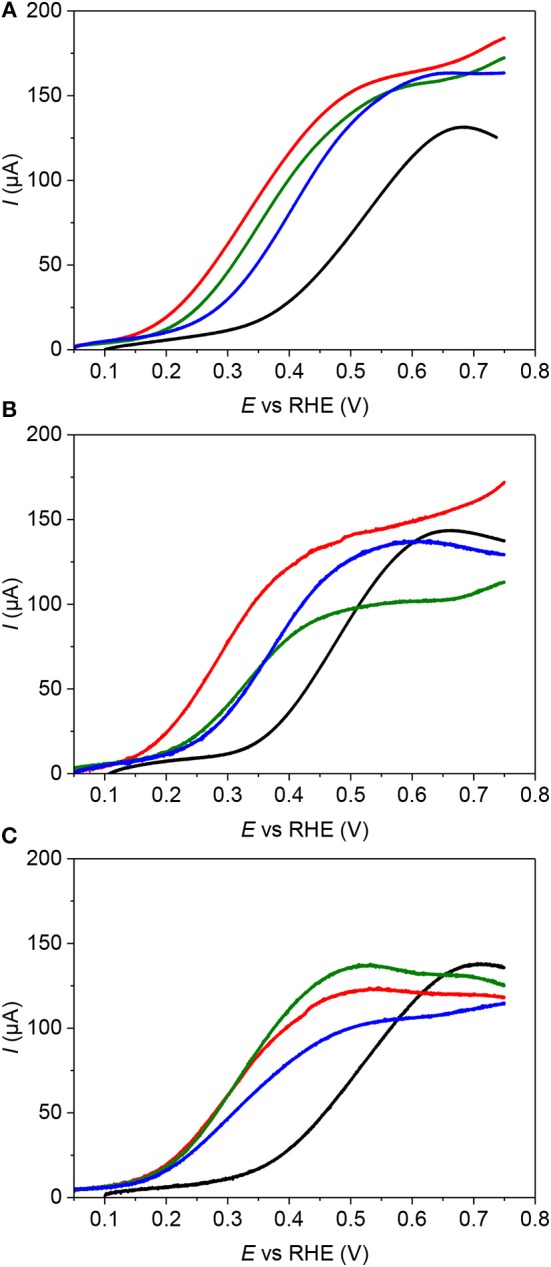
Polarization curves recorded for the electrooxidation of glucose 10^−1^ mol L^−1^ in NaOH 10^−1^ mol L^−1^ aqueous electrolyte (T = 293 K, scan rate = 5 mV s^−1^) on Au-modified Pd-NPs by dipping 1 min in HAuCl_4_ solutions. **(A)** Pd-NS, **(B)** Pd-NC, and **(C)** Pd-NO. – for pure Pd-NPs, – after dipping 1 min in HAuCl_4_ 0.01 M, – after dipping 1 min in HAuCl_4_ 0.10 M and – after dipping 1 min in HAuCl_4_ 1.00 M.

**Figure 6:**
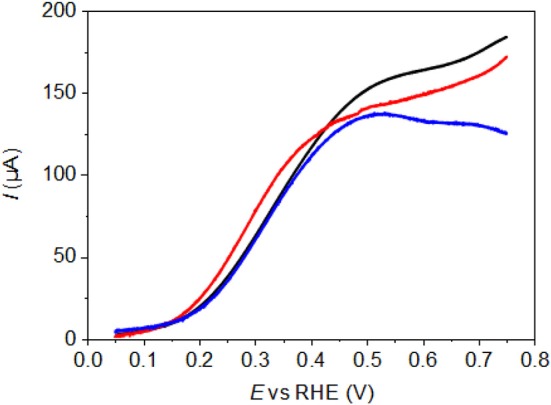
Polarization curves recorded for the electrooxidation of glucose 10^−1^ mol L^−1^ in NaOH 10^−1^ mol L^−1^ aqueous electrolyte (T = 293 K, scan rate = 5 mV s^−1^) on Au-modified Pd-NPs after dipping 1 min in 1.00 M HAuCl_4_ solution. – for Pd-NS, – for Pd-NC and – for Pd-NO.

Figure 5 compares the polarization curves recorded after modification of the Pd-NPs surfaces by dipping 1 min in solutions of 0.01 M, 0.10 M, and 1.00 M HAuCl_4_. The first remark is that the modification of the Pd surface greatly enhances the activity at low potentials, as higher current densities are observed from 0.150 V vs. RHE to ca. 0.600 V vs. RHE for all electrodes. The trend seems to be that the higher the gold coverage is, the higher the activity. This results is in agreement with previous results we obtained for the oxidation of glucose on carbon-supported Pd_x_Au_10−x_ alloys showing that the lower onset potential and the higher activity were achieved with Pd_3_Au_7_/C and Pd_1_Au_9_/C (atomic compositions) displaying global atomic surface ratios of Pd 28 %/ Au 72 % and Pd 22 %/Au 78%, respectively (Rafaïdeen et al., 2019). Now comparing the polarization curves for the highest Au coverages of Pd-NPs obtained with 1 min dipping of the electrode in 1.00 M HAuCl_4_ solution, it appears that Pd-NCs still display the higher activity (Figure 6), whereas Pd-NOs and Pd-NSs lead to the same activity between 0.150 and 0.400 V vs. RHE.

Here it is difficult to discard the effect of the surface alloy to that of non-alloyed gold nanostructures on Pd-NPs toward the glucose oxidation reaction. For the low HAuCl_4_ concentration (0.01 M), the formation of PdAu surface alloys is evidenced by the shift of the reduction peak position, whereas no reduction peaks related to pure Au structures could be detected. All modified Pd-NPs led to a significant shift of glucose oxidation polarization curves toward lower potentials, which seems to indicate that the formation of surface PdAu alloys allows increasing the activity at low electrode potentials. For higher HAuCl_4_ concentrations, the increase of the Au content in the surface alloys seems to improve the catalytic activity of Pd-NPs. But in the same time, an increase of the pure gold electrochemical surface area occurs also, and these pure gold structure may also play a role in the whole activity improvement. However, comparing results from previous works on glucose electrooxidation at PdAu/C catalysts, the shift toward lower onset potentials of the glucose electrooxidation was found much higher in the case of alloyed PdAu materials (Rafaïdeen et al., 2019) than in the case of non-alloyed (mixture of Pd and Au nanoparticles) materials (Yan et al., 2014), which seems to indicate that surface alloys play really an important role in the catalytic activity.

**Figure 7:**
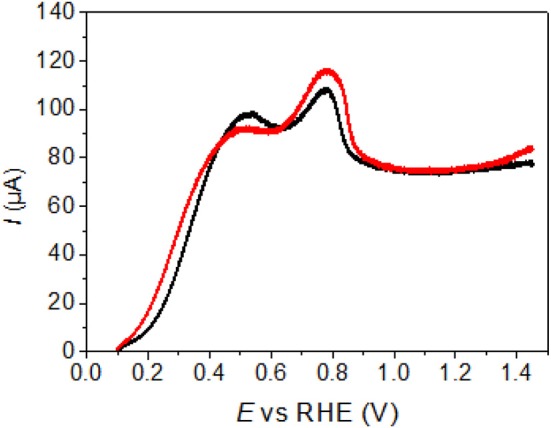
Polarization curves recorded for the electrooxidation of glucose 10^−1^ mol L^−1^ in NaOH 10^−1^ mol L^−1^ aqueous electrolyte (T = 293 K, scan rate = 5 mV s^−1^) on Au-modified Pd-NCs after dipping 1 min in 1.00 M HAuCl_4_ solution. – after cycling up to 1.45 V vs. RHE in 0.5 M H_2_SO_4_ electrolyte and – without preliminary cycle (pristine electrode).

To further investigate the surface nature of the electrocatalysts, polarization curves for glucose oxidation recorded on Pd-NCs modified by Au spontaneous deposition (1 min, 1.0 M HAuCl_4_) without cycling and after cycling up to 1.45 V vs. RHE, as example, are compared in Figure 7. The catalyst cycled up to 1.45 V vs. RHE displays slightly lower activity as the polarization curve is slightly shifted toward higher potentials. The difference in polarization curves could arise from two phenomena: (i) the formation of alloys after cycling up to 1.45 V vs. RHE or (ii) the surface orientation degradation promoted by oxygen place exchange reaction during Pd surface oxidation. But, the invariance of the peaks position speaks rather for a similar surface structure for both electrodes. Moreover, because alloyed PdAu catalysts present higher activity than non-alloyed catalysts, it is reasonable to assume that a slight surface orientation degradation due to Pd surface oxidation is responsible of the shift of the polarization curve toward higher potentials, rather than the formation of PdAu surface alloys (which should shift the polarization curves toward lower potentials). Hence this experiment speaks up for the formation of PdAu surface alloys during gold deposition process.

Anyway, this study clearly shows that the modification of Pd-NPs by spontaneous gold deposition leads to improve the catalytic activity of palladium nanoparticles. On the one hand, the Pd-NP shape is important because without Au surface modification, a lower onset potential for glucose electrooxidation is recorded on Pd-NC than on Pd-NOs and Pd-NSs. On the other hand, the modification of Pd surfaces by Au leads also to shift the onset potential toward lower values. The combination of both effects, shape and Au surface modification, makes Au-modified Pd-NCs the most active catalyst of the series for glucose electrooxidation.

## Conclusion

The syntheses of Pd-NPs with controlled size- and shape-distribution, as determined by TEM/HRTEM, have been successively carried out. Electrochemical investigation in acidic medium confirmed the preferential (100) surface orientation for Pd-NC material, (111) surface orientation for Pd-NO material and the absence of preferential orientation for Pd-NS material. The comparison of polarization curves recorded on pure Pd-NPs for the electrooxidation of glucose in alkaline medium showed that the activity depended on the main surface domains exhibited by the Pd-NPs: Pd-NC catalyst with mainly (100) surface domains displayed higher activity than Pd-NO catalyst with mainly (111) surface domains and Pd-NS catalyst without preferential surface domains. Pd-NO and Pd-NS electrodes led to the same activity, which evidenced clearly that (100) domains are the most active in a potential range higher than 0.300 V vs. RHE. The controlled modification of Pd surfaces has been carried out by spontaneous gold adsorption in order to control the surface coverage. The following observations were made: (i) for low Au coverage, the spontaneous deposition of Au on Pd-NPs leads to the formation of surface structure with surface alloy behaviors, (ii) for high Au coverage, both alloy structures and non-alloyed gold structures are formed at the surface of the Pd-NPs, (iii) for high Au coverage, two PdAu surface alloys with different compositions co-exist, the first one with the Pd_86_Au_14_ atomic composition, the second being gold-richer, (iv) the modification of Pd-NPs by gold increases the activity toward glucose oxidation, the Au-modified Pd-NC electrodes remaining the most active and (v) although it seems that the formation of PdAu alloy-like structures at the Pd-NPs surfaces has an important effect on the catalytic activity toward glucose electrooxidation, it is difficult to discard its role on the global electroactivity enhancement from that of non-alloyed Au structure on the Pd-NPs.

Although these results shed light on the surface structure of Pd-Au systems at the nanoscale, the relationship between this surface structure and the electrocatalytic behavior has still to be better understood. Indeed, the establishment of a material structure/electrocatalytic behavior relationship is essential on a fundamental point of view for the determination of reaction mechanisms and on a practical point of view for the rational design of active and selective catalysts toward aldose oxidation. For this purpose, the development of a method for the quantitative determination of the (100) and (111) surface domain ratios on Pd-NPs and deeper investigations on the kinetics of the formation of PdAu surface alloys are first necessary. These investigations will allow fixing the experimental parameters to synthesize well-characterized Pd-NPs modified by only PdAu surface alloys of controlled compositions, and/or by PdAu surface alloys of controlled compositions together with non-alloyed Au, and further to propose a more complete structure/electrocatalytic behavior relationship.

## Data Availability

The raw data supporting the conclusions of this manuscript will be made available by the authors, without undue reservation, to any qualified researcher.

## Author Contributions

All authors listed have made a substantial, direct and intellectual contribution to the work, and approved it for publication.

### Conflict of Interest Statement

The authors declare that the research was conducted in the absence of any commercial or financial relationships that could be construed as a potential conflict of interest.
